# Effects of Lifestyle and Environmental Factors on the Risk of Acute Myeloid Leukemia: Result of a Hospital-based Case-Control Study

**DOI:** 10.34172/jrhs.2021.58

**Published:** 2021-08-12

**Authors:** Masumeh Maleki Behzad, Mohammad Abbasi, Iman Oliaei, Somayeh Ghorbani Gholiabad, Hassan Rafieemehr

**Affiliations:** ^1^Blood Transfusion Research Center, High Institute for Research and Education in Transfusion, Hamadan, Iran; ^2^Department of Internal Medicine, Faculty of Medicine, Hamadan University of Medical Sciences, Iran; ^3^Students Research Center, Hamadan University of Medical Sciences, Hamadan, Iran; ^4^Department of Biostatistics, Faculty of Public Health, Hamadan University of Medical Sciences, Iran; ^5^Department of Medical Laboratory Sciences, Faculty of Paramedicine, Hamadan University of Medical Sciences, Hamadan, Iran

**Keywords:** Acute Myeloid Leukemia, Epidemiology, Risk Factors

## Abstract

**Background:** Acute myeloid leukemia (AML) is a common malignancy in adults. A vast variety of environmental and lifestyle factors play a role in AML incidence. This study aimed to assess the factors related to AML.

**Study design:** A case-control study.

**Methods:** This case-control study was performed on 137 AML cases during 2018-2021 at Beheshti Hospital in Hamadan, Iran, and 137 gender/age-matched controls. A questionnaire including 12 items was used to obtain information about lifestyle and environmental factors. A univariate and multiple variate logistic regression was used to estimate the odds ratios (OR), and a 95% confidence interval (CI) was used to investigate the relationship between the studied variables and the incidence of AML.

**Results:** Based on findings, 62 (45.3%) out of the 137 leukemic cases were male and 75 (4.7%) were females. A statistically significant increased risk for AML was found with regard to prior usage of cytotoxic agents (OR: 8.00, 95% CI: 1.01, 63.9, *P*=0.050), family history of malignancies (OR: 3.62, 95% CI: 1.65, 7.92, *P*=0.001), exposure to electrical power (OR: 3.22, 95% CI: 1.52, 6.81, *P*=0.002), and history of mental diseases (OR: 8.50, 95% CI: 3.64, 19.80, *P*=0.001). It was found that the AML incidence had no association with age, gender, radiation therapy, cigarette smoking, prior chemotherapy, congenital disorders, exposure to chemical agents, history of infectious mononucleosis, exercise, and blood transfusion (*P*>0.05).

**Conclusion:** The current results suggested that cytotoxic agents, family history of malignancy, mental disorders, and exposure to electrical power could play a role in AML incidence.

## Introduction

Acute myeloid leukemia (AML) is the most common hematological malignancy in adults^1^. Rapid growth of abnormal myeloid progenitor cells in the bone marrow (BM) and peripheral blood (PB) is the most important sign of AML^2^. According to reports by the World Health Organization, the incidence of AML is related to several chromosomal abnormalities that have similar clinical manifestations but different morphologic, immunophenotypic, and cytogenetic subtypes^3^. Moreover, altered expression of a large number of genes, including cytokine, ion channel, as well as CD markers may play a role in the pathogenesis and prognosis of AML^4-7^.

Besides the chromosomal abnormalities, epidemiologic studies demonstrated that several factors, including exposure to ionizing radiation, personal and family medical histories, exposure to chemical or biological agents, such as solvents and benzene, smoking habits, and lifestyle factors are associated with AML and acute lymphoid leukemia (ALL) incidence^2,8-10^. Furthermore, it has been shown that the process of aging can be associated with an increased risk of AML that is likely due to the gradual deterioration of the immune system due to immunosenescence^11^. It seems that lifestyle and educational intervention play a key role in the promotion of community health^12,13^. Studies have also highlighted the impact of chemotherapeutic agents on AML incidence. For example, it has been found that older patients with AML have a history of prior chemotherapy exposure^14^. This may be a result of BM niche alteration that leads to deregulation of balance within the marrow cavity as well as abnormal hematopoiesis^15^.

The specific genetic abnormalities that cause AML are not usually modifiable^16^. However, the majority of epidemiologic studies that have demonstrated relationships of AML incidence with potential risk factors, such as exposure to ionizing radiation and chemicals, including hydrocarbons and pesticides, cigarette smoking, infections, and family history of malignancies, have shown that some of these factors are simply modifiable^17,18^. In addition, it seems that these potentially etiologic exposures have an impact on clinical outcomes. Therefore, understanding relevant risk factors may have particular significance in the prevention and reduction of AML occurrence. Moreover, it should be mentioned that this may influence clinical decisions.

It is noteworthy that since the development of leukemia takes place sometimes years after environmental exposure, current knowledge of some risk factors is insufficient. For example, in utero exposure to ionizing, radiation is considered an established cause of pediatric de novo AML^19^; nevertheless, as the disease occurs sometimes years after the exposure, this risk factor is not usually considered. However, since leukemia develops sometimes years after environmental exposure, current knowledge of some risk factors is insufficient.

 Furthermore, few studies have been conducted to examine the possibility of synergistic interactions of genes and environmental factors with AML both during childhood and adulthood. It seems that the knowledge of AML etiology can be enhanced by exploring the potential effect of several environmental and lifestyle factors, namely diabetes, exposure to alcohol, and pesticides as well as maternal factors, including age at pregnancy and birth weight on genetic abnormalities.

 In spite of the high incidence rate of AML due to environmental risk factors, few studies in Iran explain the majority of risk factors. In the present case-control study, an extensive epidemiological analysis of AML was conducted based on personal characteristics, lifestyle, and other environmental risk factors, including occupations and specific exposures to the chemical agents.

## Methods

**Figure 1:**
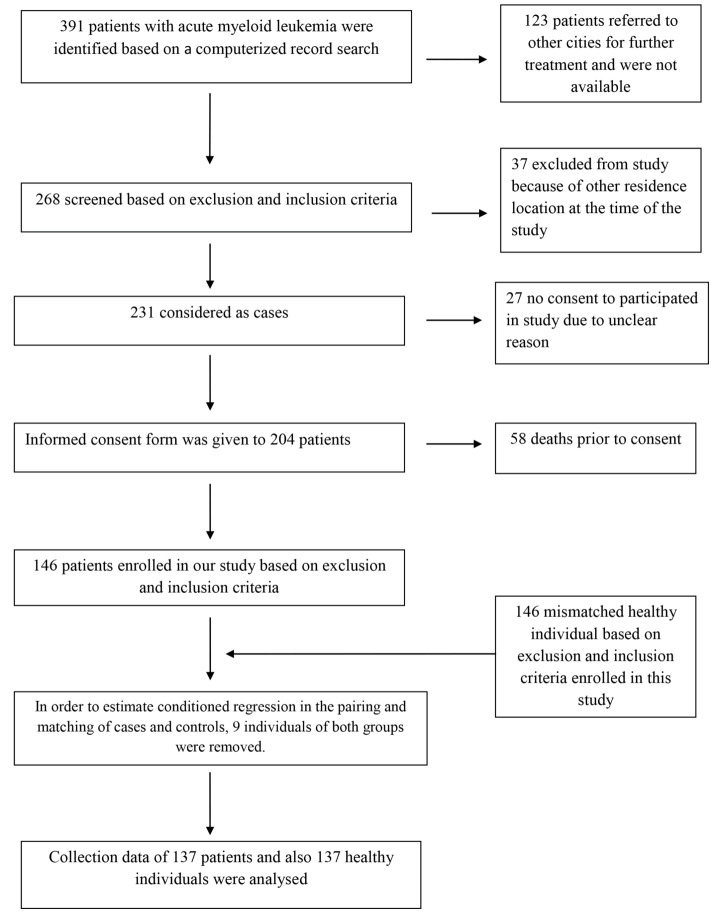
Study flow chart of the selection of subjects.

This matched case-control study was conducted on patients with AML who referred to Beheshti Hospital of Hamadan University of Medical Sciences, Hamadan, Iran. For the purposes of the study, 137 patients were selected from 391 newly diagnosed (confirmed through BM aspiration as well as flow cytometric analysis of the PB and BM cells), relapsed, or refractory AML patients who were hospitalized during 2018-2021 (Figure 1). In addition, one healthy matching control group was added to the study, including 137 people who were randomly selected from individuals who referred to the same hospital for routine healthcare.

 The control group matched the case group by age (±5 years) and had resided in Hamadan during the last 20 years. If the control group members refused participation after enrolment or were not eligible, additional subjects were randomly selected. Control group members were also screened to make sure that they were not relatives of the case group members.

 Data collection from both case and control groups began in February 2018 and finished in April 2021. It should be noted that the matching criteria included gender and age. Moreover, inclusion criteria for cases were diagnosis of AML and residence location at the time of the study. The inclusion criteria for controls were no previous history of invasive cancers or hematological malignancies and residence in Hamadan at the time of the study. The exclusion criteria for cases and controls were unwillingness to participate in the study, other resident location at the time of the study, and lack of access to patients. Informed consent was obtained from each subject in this study before data collection. Detailed information on the variables under study was collected through a full questionnaire that was given to each participant of the study. The questionnaire included items about age, gender, and a large number of potential risk factors, such as personal and family medical histories (e.g., history of hematological malignancies or solid tumors in the family, Down syndrome, Kostmann syndrome, aplastic anemia, mental diseases [i.e., sadness, anxiety, depression, fear, stress, and hopelessness], blood transfusion history, chemotherapeutics agents for cancer treatment, X-ray) exposure, and lifestyle, including smoking (>100 cigarettes in a year) and exercise, environmental exposures, such as living on a farm, living within 100 meters of high voltage electrical power transmission lines for at least five years, and exposures to chemicals (only daily or weekly exposure to chemical agents was considered exposure and low dose exposures and occasional ones were not; like once per every few months or every one year), physical or biological agents (e.g., benzene, solvents, oil, radiation, and viruses, such as Epstein–Barr virus [EBV] or infectious mononucleosis).

###  Statistical analysis

The demographic data were presented as frequency and percentage while the quantitative data were expressed as mean ±SD. Relevant data were collected in Stata software (version 14.2) and the risk factors were determined in two ways. In the first method, the conditional logistic regression of univariate analysis was used with the assumption of individual matching while in the second method, the multiple conditional logistic regression analysis was employed. The odds ratios and a 95% confidence interval were used to investigate the relationship between the studied variables and predisposition to AML. The chi-square and t-tests which showed good results in all models were used to compare the case and control groups to determine the association between the studied variables and the risk of AML. It should be mentioned that a *P-*value of less than 0.05 was considered statistically significant. All statistical analyses were performed using SPSS software (version 22) and STATA (version 14.2).

## Results

**Figure 2:**
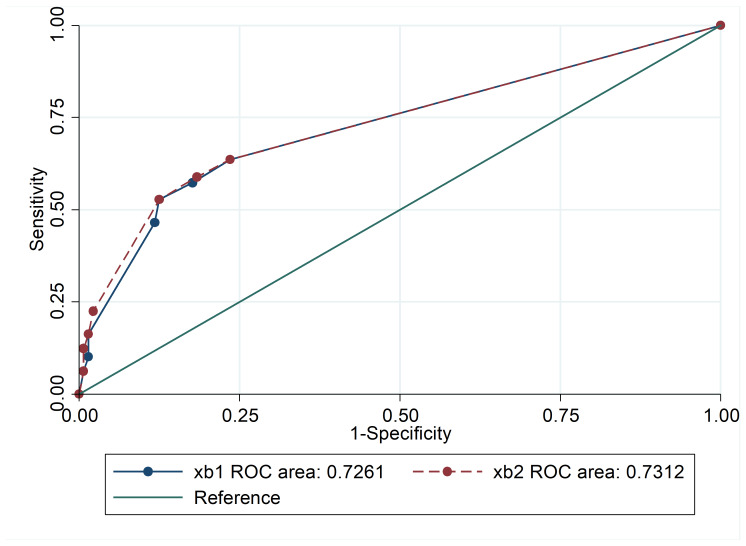
The area under the receiver operating characteristic (ROC) curve, the sensitivity, and specificity of the multivariate logistic regression models (conditional and unconditional).

A total of 137 patients with AML and 137 age/gender-matched healthy individuals as the controls group were included in the study analysis. There was no significant difference between the mean ages of both groups (*P*=0.055) and almost homogeneity was seen in the distribution. As shown in the receiver operating characteristic diagram, no significant difference was observed between the goodness of fit criteria of the two models (Figure 2). In total, 62 (45.3%) of the leukemic cases were male and 75 (54.7%) were female. As shown in Table 1, the mean age of AML patients was 50.86±18.6 years (range: 16-83) while the mean age of the control group was 46.81±19.5 years (range: 4-79). Due to the successful matching, there were no differences between cases and controls with respect to age (*P*=0.08) and gender (*P*=0.45).

To identify the AML risk factors, the association between different variables and AML incidence univariate analysis of risk factors was examined using conditional logistic regression (Table 1). On the basis of the calculations of population-attributable risk factors, some were identified as significant in our analyses. A significant association was found between prior usage of cytotoxic agents (*P*=0.05) and increased risk of AML (Table 1).

**Figure 3:**
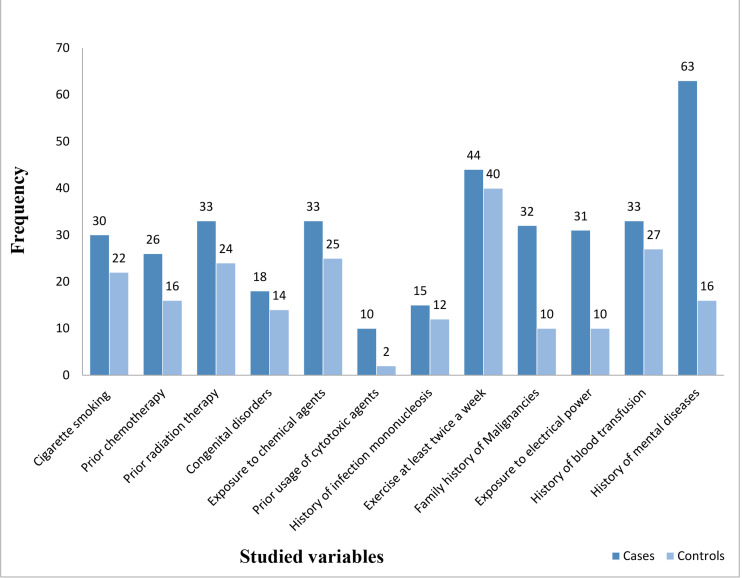
Comparison of studied variables between case and control groups.

Moreover, the univariate analysis by conditional logistic regression showed that the odds of developing AML for individuals with a history of using cytotoxic agents were eight times higher than others (95% CI: 1.01, 63.9). Family history of hematological malignancies or solid tumor (*P*=0.001) and history of mental diseases (*P*=0.001) were also associated with an increased risk of AML (Figure 3). In this regard, the univariate analysis by conditional logistic regression showed that the odds of people with a family history of malignancies and mental diseases for developing AML were 3.62 and 8.5 times higher than others, respectively (95% CI: 1.65, 7.92), (95% CI: 0.3.64, 19.8).

**Table 1:** Univariate analysis of risk factors for acute myeloid leukemia by using conditional logistic regression.

**Variables**	**Cases**	**Controls**	**Odds Ratio** **(95% CI)**	***P*** **-value**
Cigarette smoking				
No	107	115	1.00	
Yes	30	22	1.50 (0.79, 2.82)	0.209
Prior chemotherapy				
No	111	121	1.00	
Yes	26	16	1.76 (0.89, 3.49)	0.101
Prior radiation therapy				
No	104	113	1.00	
Yes	33	24	1.36 (0.78, 2.36)	0.269
Congenital disorders				
No	109	123	1.00	
Yes	18	14	1.60 (0.72, 3.52)	0.244
Exposure to chemical agents		
No	104	112	1.00	
Yes	33	25	1.58 (0.86, 2.91)	0.135
Prior usage of cytotoxic agents		
No	127	135	1.00	
Yes	10	2	8.00 (1.01, 63.96)	0.050
History of infectious mononucleosis		
No	122	125	1.00	
Yes	15	12	1.27 (0.57, 2.80)	0.549
Exercise at least twice a week		
No	93	97	1.00	
Yes	44	40	1.06 (0.64, 1.75)	0.801
Family history of malignancy or solid tumors	
No	105	127	1.00	
Yes	32	10	3.62 (1.65, 7.92)	0.001
Exposure to electrical power		
No	106	127	1.00	
Yes	31	10	3.22 (1.52, 6.80)	0.002
History of Blood transfusion		
No	104	110	1.00	
Yes	33	27	1.21 (0.65, 2.22)	0.538
History of mental diseases			
No	74	121	1.00	
Yes	63	16	8.50 (3.64, 19.80)	0.001

CI: confidence interval

As seen in Table 1, there was a significant correlation between the risk of AML and exposure to electrical power in conditional logistic regression analysis (*P*=0.002). People with exposure to electrical power had 3.22 times higher odds for AML occurrence, compared to others (95% CI: 1-52, 6.81). In addition, the risk of AML was shown to be 1.76 times higher for patients with prior chemotherapy, although no statistical significance was observed (*P*=0.101), (95% CI: 0.89, 3.49). Furthermore, it should be noted that no association was found between smoking and the risk of AML incidence (*P*=0.209). However, the univariate analysis of cigarette smoking showed that people with a history of smoking had 1.5 times higher odds for AML incidence in comparison to others.

In addition, AML incidence had no association with prior radiation therapy, congenital disorders, exposure to chemical agents, history of infectious mononucleosis, history of blood transfusion, and exercise (Table 1). To investigate the simultaneous effect of the variables and eliminate possible confounding variables, the variables with a significance level of less than 0.1 in each of the above models simultaneously were entered in the multivariable conditional logistic regression models. The Backward Stepwise method was used to remove the distorters and adjust significant findings. Based on the results of the analysis of multivariate conditional logistic regression models, a history of mental diseases has a significant effect on AML incidence (*P*=0.001) (Table 2).

**Table 2:** Adjusted analysis of risk factors for acute myeloid leukemia by using conditional multiple logistic regression.

**Variables**	**Cases**	**Controls**	**Odds Ratio (95% CI)**	***P*** **-value**
Family history of hematologic malignancy or solid tumors				
No	105	127	1.00	
Yes	2	10	2.21 (0.89, 5.48)	0.085
Exposure to electrical power				
No	106	127	1.00	
Yes	31	10	2.26 (0.90, 5.66)	0.080
History of mental diseases				
No	74	121	1.00	
Yes	63	16	7.79 (3.23, 18.77)	0.001

CI: confidence interval

Results of the univariate analysis showed a significant relationship between family history of malignancies and AML incidence; however, this relationship was not observed in the results of multivariable conditional regression analysis (*P*=0.085). Moreover, the results of the multivariate analysis did not show a significant relationship between exposure to electrical power and AML incidence in conditional logistic regression (*P*=0.080) (Table 2).

## Discussion

The AML and other hematological malignancies may be the result of a combination of genetic abnormalities and environmental risk factors^20,21^; hence, it can be said that the etiology of AML is complex. Several epidemiologic studies worldwide have demonstrated that AML incidence is associated with a number of possible risk factors, including infectious and environmental factors, such as ionizing radiation, cigarette smoking, history of malignancies, and drug use^17,20^. In this case-control study, the association between environmental risk factors and the development of AML has been evaluated. In the present study, results of the univariate and multivariate analysis indicated that AML can be related to a family history of malignancies, exposure to electrical power, and mental disorders.

 This study was performed on 137 cases and the same number of healthy controls, and it was found that family history of malignancies may be associated with AML incidence. Although only a small number of cases and controls had a family history of malignancies in this study, a significant association was observed between family history of hematological malignancies and solid tumors with the incidence of AML.

In their case-control study, Wong et al. reported only the weak and non-significant effect of family history of malignancies on AML ^8^ which is inconsistent with the findings of the present study. Other studies have demonstrated that the family history of cancer has a relative risk for incidence of AML and different neoplasms^22,23^ which is in line with the findings of this study. However, it should be mentioned that this finding was not replicated in a case-control study carried out in United States^24^.

These findings suggest that studies should determine whether AML patients with a family history of malignancies have received benefit healthcare from an earlier age or not as healthcare utilization can be useful in cancer prevention. Moreover, it is obvious that the development and progression of cancer usually occur due to the abnormal expression of some genes, such as tumor suppressor^25,26^. Since for the expression of a trait each allele is inherited from one of the parents, it seems that the acquisition of abnormal genes, as well as the incidence of cancer, is higher in populations with high rates of consanguineous marriage. Therefore, consanguineous marriage should be re-examined in populations where leukemia and other tumors seem more common.

In the present study, the analysis showed a significant relationship between exposure to electrical power and risk of AML incidence. In contrast, in a case-control study performed by Wong et al., living within 100 m of high voltage electrical power transmission lines was not found to be associated with the risk of AML incidence^8^. However, there are many studies that have found the relationship between exposure to electrical power and a large number of disorders, including cancer^27^, genetic damage^28^, reproductive disorders^29^, immune dysfunction^30^, kidney damage^31^, and hypersensitivity^32^. Since the progress of science and technology leads to the wide use of high voltage electrical power transmission lines, electromagnetic wave exposure has become a public health problem that requires public awareness.

In the present study, an increased risk of AML was observed in approximately half of the cases who had a history of mental diseases, such as sadness, anxiety, depression, fear, and stress. It has been shown that the stress from the family is the most important source of stress associated with adolescent mental health^33^. No study has been conducted to explore the direct impact of mental disorders, including anxiety and depression, on AML incidence. Accordingly, it is unclear why mental diseases are a risk factor for the development of AML. Ding et al. in their case-control study demonstrated that anxiety and depression have poor prognostic value for survival in AML patients^34^; hence, mental diseases which are associated with anxiety and depression might play a role in AML incidence via an unfavorable effect on the quality of life.

Generally, smoking has been recognized as a risk factor for AML but in contrast to previous studies, we did not find any significant association between smoking and AML incidence. Results of a case-control study carried out by Bjork et al. indicated no significant association between smoking and AML incidence^35^; this is in line with the findings of the present study. However, many case-control studies have indicated that the increased risk of AML is associated with heavy smoking^9,36,37^. In addition, findings of a large cohort study performed by Ma et al. demonstrated that smokers who smoked more than a pack per day had a statistically increased risk of AML^38^.

Our non-significant results in this regard may stem from a lack of AML classification or exact duration of smoking in our cases. Accordingly, Wong et al. in a case-control study found that the number of daily consumed cigarettes had a non-significant association with AML, whereas the duration of smoking seemed to be positively associated with the risk of developing AML^8^. However, inconsistencies in definitions and measurements of this variable in different studies make it difficult to interpret the full impact of smoking on AML.

In this study, no clear relationship was found between a history of prior chemotherapy and AML. Despite this lack of association in the present research, the incidence of AML after chemotherapy is a well-established fact^39-41^. It should be mentioned that the sample size of the present study was small (n=25); therefore, there is a need for further clinical studies with larger sample sizes. The leukemogenic effect of chemotherapy could be due to the fact that its purpose may be BM progenitor cells. Many models are available to estimate the risks of AML after chemotherapy; however, to our knowledge, no tools exist to reliably recognize what patients are at risk. Nevertheless, the use of BM and PB cytogenetic assessment and a more careful clinical follow-up is an important prognostic tool for awareness of the risk of AML.

In the present study, no significant association was observed between prior radiation therapy and AML. In line with our results, those of several case-control studies have not shown the increased risk of leukemia in children who were exposed to X-rays during the postnatal period ^42,43^. However, Ojha et al. found an increased risk for AML due to radiotherapy secondary to benign diseases ^44^. These inconsistent results may be in part due to differences in the methods used for the assessment of radiation exposures (exposure to three or more rays) or the age of the studied population. Although there are heterogeneous results for the relationship between exposure to radiation and leukemia in previous studies, this risk appears to be associated with therapeutic radiation effects on DNA repair enzymes, such as topoisomerase II. In this regard, Leone et al. have shown that alkylators combined with radiotherapy could promote survival of misrepaired cells giving rise to the leukemic clone^45^.

Potential exposures to a variety of chemicals, such as paints, hydrocarbons, including benzene, chemical fertilizers, detergents, and pesticides, might have been potentially hazardous for AML incidence. Prior case-control studies have identified an association between chemical exposure, especially exposure to benzene, and increased risk of AML^1,46,47^; however, in this study, no significant association was observed. Despite our negative results and similar findings of a case-control study performed by Poynter et al. on pesticides and agricultural chemicals exposure in Minnesota^48^, it seems that any chemical exposure during any time period of life in houses or workplaces could be a potential risk factor for cancers. Therefore, these public health problems should be considered and public awareness should be raised more widely.

In this study, no significant associations were observed regarding the effects of congenital disorders, such as Down syndrome, Kostmann syndrome, and aplastic anemia on AML. There is small speculation about the possible effects of congenital disorders on AML. Although adults with Down syndrome show a decreased incidence rate of cancer, compared to healthy individuals, children with Down syndrome are at an increased risk of leukemia, especially AML^49^. Future studies should focus on age-stratified approaches to reach results with greater sensitivity.

Besides, when we evaluated the relationship between exercises at least twice a week with AML, we did not observe a significant association. Since several quasi-experimental studies have indicated that physical activity and aerobic exercise interventions have been helpful for the reduction of fatigue and improvement of the quality of life of leukemic patients^50-52^, we believe that exercise can improve immune system function and increase the ability to fight diseases.

The EBV is a ubiquitous human infection that has been associated with a variety of malignancies, such as Burkitt’s lymphoma (BL), Hodgkin lymphoma (HL), nasopharyngeal carcinoma (NPC), gastric cancer, and acute leukemia^53^. The current study showed evidence of EB infection in a very small number of AML patients and no significant association was found between them. The findings are inconsistent with those of previous studies that indicated EBV infection as a possible risk factor for the progression and poor clinical outcome of chronic lymphoblastic leukemia(CLL), ALL, and AML^54,55^. Since we do not have enough evidence to conclude that EBV is associated with the pathogenesis of AML, it is suggested that further studies be conducted with larger sample sizes of patients to investigate whether there is a relationship between EBV and AML.

In addition, our study showed no association between a history of blood transfusion and AML incidence. Similar to our results, in the two US population‐based case‐control studies, the increased risk of hematological malignancies was not associated with blood transfusion^56,57^. However, a slightly higher risk of leukemia (all types) was reported in individuals with a history of blood transfusion in a study performed by Ross et al. ^58^. Since the relationship between the history of blood transfusion and leukemia is a controversial issue, further studies should be performed on specific leukemia subtypes are required. There were also several limitations in the present research; first of all, this study was conducted in a single center. Moreover, there was a lack of identification of several cytogenetic groups. Furthermore, regarding the history of mental diseases, only anxiety and depression were included; however, it is better to include other mental disorders as well. Besides, the duration of the study was relatively short. Therefore, it is suggested that the results be validated in a long-duration study in the future.

## Conclusion

 In summary, it was found several potential risk factors of AML (all subtypes combined) include prior use of cytotoxic agents, family history of hematologic malignancy or solid tumors, and history of mental diseases. An unexpected finding in this study was the lack of associations of cigarette smoking and prior radiation therapy with increased risk of AML. Identification of AML risk factors may be helpful to improve therapeutic measures and public health protocols. However, the limitations of the study should be taken into consideration. First, because of the large number of risk factors in AML subtypes, only some of them were evaluated in this study. Second, the number of subjects in this study was limited; hence, the results need to be interpreted with caution. Finally, the findings need to be replicated in future studies to reach definitive results.

## Acknowledgments

 The authors wish to thank all their colleagues at Hamadan University of Medical Sciences.

## Conflict of interests

 All authors approved the manuscript and have no declaration regarding commercial or non-commercial intentions.

## Funding

 This study was funded by Research and Technology Deputy, Hamadan University of Medical Sciences (No. 9709065171).

## Authors’ Contributions

 H.R. conceived the manuscript and revised it. M.A provided clinical data and information. M.M.B, I.O., and S.G performed the statistical analysis, wrote the manuscript, and prepared tables and figures.

## Ethical Considerations

 All procedures were approved by the institutional Ethics Committee of Research Deputy of Hamadan University (IR.UMSHA.REC.1397.558). Accordingly, informed consent was obtained from all the participants in the study.

## Highlights

- Acute myeloid leukemia may be the result of a variety of environmental risk factors.
- Family history of malignancies, environmental exposure, and history of mental diseases may be a potential risk for acute myeloid leukemia.
- Identification of these risk factors may be helpful in the management of patients as well as improvement of public health protocols.
